# Cellular and Virtualization Technologies for UAVs: An Experimental Perspective

**DOI:** 10.3390/s21093093

**Published:** 2021-04-29

**Authors:** Victor Sanchez-Aguero, Luis F. Gonzalez, Francisco Valera, Ivan Vidal, Rafael A. López da Silva

**Affiliations:** 1 IMDEA Networks Institute, Avda. del Mar Mediterráneo, 22, 28918 Madrid, Spain; 2 Department of Telematic Engineering, University Carlos III of Madrid, 28911 Leganes, Spain; luisfgon@it.uc3m.es (L.F.G.); fvalera@it.uc3m.es (F.V.); ividal@it.uc3m.es (I.V.); 3 Telefónica I+D, Distrito Telefónica, Ronda de la Comunicación s/n, 28050 Madrid, Spain; rafaelalejandro.lopezdasilva@telefonica.com

**Keywords:** UTM, cellular-connected, UAV, UAS, testbed, BRLoS, virtualization

## Abstract

The Unmanned Aircraft System (UAS) ecosystem is exponentially growing in both recreational and professional fields to provide novel services and applications to consumers from multiple engineering fields. However, this technology has only scraped the surface of its potential, especially in those cases that require fast reaction times. Accordingly, the UAS Traffic Management (UTM) project aims at efficiently managing the air traffic for Unmanned Aerial Vehicle (UAV) operations, including those cases where UAVs might be remotely managed from a completely different geographical location. With these considerations in mind, this article presents a cellular-assisted UAVs testbed used to complete a mission managed beyond the radio line-of-sight (BRLoS), as well as introducing a virtualization platform for deploying services using containerization technology. In addition, the article conducts a communication performance evaluation in order to determine if the testbed equipment meets the requirements to carry out this BRLoS management. Finally, indoor flight operations are carried out to demonstrate the feasibility and proper operation of the testbed.

## 1. Introduction

Unmanned Aerial Vehicle (UAV) operations have been exponentially growing over the last few years, not only for non-professional users (e.g., recreational flights, aerial photography, racing competitions), but also for commercial/professional purposes, providing a wide array of services in many types of environments, including both civil and military fields or even urban populated areas (e.g., packet delivery, medical supplies transportation, infrastructure surveillance, emergency response). Many attributes inherent to these aerial vehicles, such as fast mobility, flexibility, and adaptability to different conditions, enable promising wireless services and application deployments when combined with the appropriate communication facilities. UAVs have the possibility of, literally, becoming mobile terminals when connected to a conventional cellular network, which together with their capacity to carry a large variety of equipment as payloads (e.g., video cameras, sensors), allow them to potentially support several tasks such as real-time video streaming [1] or item delivery (Amazon Prime Air: https://www.amazon.com/b?node=8037720011 (accessed on 27 April 2020)), creating promising business opportunities for stakeholders and cellular operators. These operations involve a certain degree of risk due to their complexity and nature in civilian environments. Although they have proven to be of great value to society in several circumstances, such as delivering time-critical medical supplies or evaluating disaster areas, there is a significant challenge of managing all the applications simultaneously and in the same airspace.

In this context, relevant institutions such as the Federal Aviation Administration (FAA), Google, Amazon, or NASA are working together on the UAS Traffic Management (UTM) project [2,3]. Like the internationally well-established Air Traffic Management (ATM) ecosystem, the UTM’s aim is to manage automated, safe, and efficient low altitude airspace operations in UAS operations. Notwithstanding their similarities, which derive from tackling a common issue between them (controlling airspace), UTM and ATM have significant differences. The lower airspace (managed by UTM) is continuously subjected to several changes, and the situation on the ground must be continuously monitored. Besides, the trajectories calculation at lower airspace must take into account not only the collisions with other vehicles, but the ones with buildings and potential obstacles as well.

**Figure 1:**
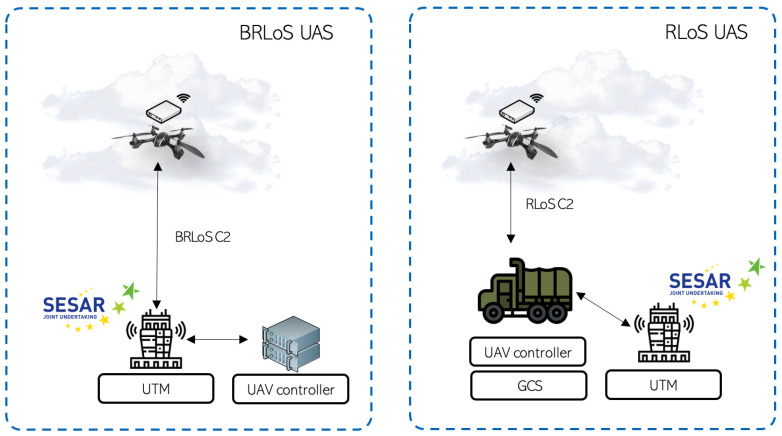
Different types of UAS following UTM: Beyond Radio LoS (BRLoS), and Radio LoS (RLoS).

UAVs are commonly controlled using Radio Line-of-Sight (RLoS) from a Ground Control Station (GCS) (i.e., a direct link from the GCS to the UAVs). In particular, Small UAVs (SUAV) must operate in a relatively close area (depending on the selected RLoS technology) to the GCS to enable the connection. In contrast, the UTM project researches (among other topics) enabling Beyond Radio visual Line-of-Sight (BRLoS) UAV operations at low altitudes (below 120 m above ground level), facilitating and boosting near-future UAV commercial applications. Figure 1 presents the RLoS and BRLoS Unmanned Aerial Systems (UAS) considered above (UASs are normally understood to be both the UAV and the GCS working together). As it can be appreciated in both systems, it is essential to communicate the UAV controller with the UTM to enable emerging applications, since different UASs may coexist in the same airspace.

Many efforts over these research lines are also being invested in by the European Union by the Single European Sky ATM Research (SESAR). The SESAR initiative is working on the development of U-space [4,5], a set of new services to uphold safe, effective, and secure access to European airspace for large numbers of UASs. In addition, many projects have been funded by the European Union to develop safe and efficient UAS traffic management. These projects include 5D-AEROSAFE (5D-AeroSafe: https://5d-aerosafe.eu/ (accessed on 27 April 2020)), DRONES4SAFETY (Drones4Safety: https://drones4safety.eu/ (accessed on 27 April 2020)), RAPID (RAPID: https://rapid2020.eu/ (accessed on 27 April 2020)) or Labyrinth (Labyrinth: http://labyrinth2020.eu/ (accessed on 27 April 2020)). This research work has been done within the framework of this Labyrinth project.

In this context, the Labyrinth project aims at creating and validating new UAV applications to enhance the safety, security, and efficiency of civil system transportation and urban areas. Labyrinth will create a centralized system able to communicate with all the UAVs located in a defined area, processing their source and destination waypoints to compute their potential paths and avoid collisions (accomplishing this with the SESAR policy). Moreover, Labyrinth will build fully-operational use cases in four relevant transportation environments: (i) road (e.g., speed control, accident management, plate identification); (ii) waterborne (e.g., seaport facilities supervision); (iii) air (e.g., bird scaring, airport facilities supervision), and (iv) emergency (e.g., provisioning of medical supplies, discover escape routes). The Labyrinth consortium includes both research institutions and industrial partners, involving relevant key-adopters such as the Spanish General Directorate of Traffic (DGT), the Municipal Emergency Assistance and Rescue Service—Civil Protection (SAMUR—Protección Civil) from Madrid, and the Port Authority of the Eastern Ligurian Sea from Italy.

This article presents as the main result a functional testbed in the context of the Labyrinth project, focusing on its communications aspects by including all the required ingredients to complete a UAV mission following the U-space requirements. For this purpose, UAVs are connected to the cellular network to enable connectivity with the UAV controller (the entity in charge of computing flight trajectories and sending instructions to the UAVs) and other UAVs. Moreover, all the corresponding trajectories and instructions are sent from the UAV controller to the UAVs using an automation platform. This system runs on top of a lightweight virtualization platform to further test applications and functionalities beyond the Command and Control (C2). Besides, all this technology has been validated in an indoor flight set of experiments. This article also evaluates the 4G cellular network performance using commercial deployments with different communication prototypes and different network operators and the 5G Stand Alone (SA) cellular network in a laboratory environment. The obtained results highlight that the cellular network properties are adequate for managing the UAVs. Although the 5G commercial deployment is still imminent and does not reach a large part of the population, it serves as a proof of concept to highlight the potential of 5G networks.

The rest of the article is organized as follows: Section 2 reviews the related work and background regarding UAV technologies and communications, as well as the advances of the combination of UAV technology with virtualization. Section 3 provides details about the testbed, describing in detail all of the technologies chosen for its design, highlighting its main characteristics, functionalities, and the role each one of them in the system. Section 4 presents a set of experiments to validate the feasibility of the testbed, including a performance evaluation achieved by the communications prototype, and an indoor flight mission to demonstrate the testbed functionality. Finally, Section 5 presents the main conclusions and future work for the article.

## 2. Related Work and Background

### 2.1. Communications

Conventional research related to UAVs has typically been focused on navigation, optimal trajectories, and control challenges. Unfortunately, many articles underestimate the difficulties of maintaining stable and reliable communications between the aircrafts and the GCS, where connectivity might not be stable due to the unreliable nature of wireless communications. However, there has been a sharp increase of interest in this particular topic, as it can be seen in some examples that focus on UAS communications.

On the one hand, UAVs beyond their traditional usage as standalone mission platforms are more and more being proposed as flying wireless base stations [6,7] in 5G networks and beyond (also known as UAV-assisted cellular communications) to support network connectivity in multi-UAV missions. UAVs include different application scenarios such as coverage and capacity enhancement. UAVs can provide on-demand connectivity in events with vast amounts of people, like sport events, or concerts [8]. The authors in [9] explore the utilization of low-altitude UAVs that are provided with Base Stations (BS) to complement the terrestrial network. Aerial BSs can also assist terrestrial networks such as vehicular networks for information dissemination and connectivity improvement, thanks to their inherent mobility [10]. In [11], the authors present a UAV-assisted cellular network with optimized spectrum sharing and cyclical multiple access, which significantly improves spatial throughput over the conventional network, although the interference control complexity was higher. Other representative application is to use UAVs as BSs for public safety applications [12,13] such as natural disasters (e.g., earthquakes, typhoons, floods) management, where the conventional cellular network might be unavailable, damaged, or insufficient. Through different simulations, these articles show that the optimized deployment of aerial BSs at different areas may improve throughput coverage and efficiency. Another example is SARDO [14], a UAV-based cellular search and rescue solution (i.e., a UAV equipped with a light-weight BS) to localize missing victims through mobile phones.

On the other hand, UAVs are also being proposed as aerial user equipment (known as cellular-connected UAVs) [7,15]. Cellular-connected UAVs make use of existing cellular infrastructures and should co-exist with existing ground-based user terminals (i.e., normal users of the cellular network). This paradigm has obtained significant interest to establish reliable communications among UAVs and different devices that may be used during a complete mission (e.g., UAV-controller, UTM). Besides, considering the recent appearance of 5G networks, short term bandwidth and latency improvements are expected to be introduced once these networks have properly been deployed into the cellular landscape, which may be beneficial for multi-UAV systems for efficient fleet control [16,17]. However, the existing infrastructure is designed and optimized for terrestrial users needs, and there are still several challenges to be considered before relying on cellular-connected UAVs as a valid solution. For example, cellular-connected UAVs require high-speed uplink connectivity, while most applications for ground users demand high-speed downlink connectivity instead. Although the downlink is critical since the UAV trajectories are transmitted in this direction, most UAV applications generate information from the UAV upon the UAV-controller (e.g., video streaming, sensors), requiring higher data rates in the uplink. Moreover, UAVs might deal with significant interference introduced by the neighboring BSs because of the nonexistence of obstacles between the multiple BSs and the UAVs [18].

Finally, a pretty well established UAV paradigm for multi-UAV missions are Flying Ad hoc Networks (FANETs) [19,20]. These networks are scalable due to their facility to integrate new UAVs to the system with small deployment and maintenance costs. Another of their benefits is providing feasible communications between UAVs without any additional infrastructure, similarly to the well-known Mobile Ad hoc Networks (MANETs) or Vehicular Ad hoc Networks (VANETs). For this reason, FANETs have been the most considered solution by the research community to enable UAV communications during the last years. For example, Ref. [21] presents a FANET scenario where we evaluate a routing scheme using an emulation platform to characterize the communication channel (WiFi) and the network mobility models. Authors in [22] describe an innovative routing process based on ad hoc networks with better efficiency than traditional FANET algorithms. Another example is [23], where position-based routing algorithms are compared over a common scenario through a comprehensive comparative analysis.

### 2.2. Virtualization and UAVs

As it has been pointed out previously, UAV provide superior mobility in comparison with traditional vehicles, allowing them to implement a wide array of applications such as automatic forest fire detention mechanisms [24], warehouse transportation, or precision agriculture [25]. As a consequence, some parts of the research community have been focusing on softwarizing different applications and functionalities provided by UAVs in order to dynamically create and deploy networking services over aerial networks. Such effort will allow UASs to remove the need for specialized equipment to execute multiple services over the network, as well as reducing both development and maintenance costs. In this regard, one outstanding example of such effort can be seen in the synergetic junction of Small UAVs (SUAV) technology together with the Network Functions Virtualization (NFV) paradigm; this paradigm has risen as one of the key enablers of 5G communications, with the main objective of softwarizing physical network functions for its deployment in generic hardware equipment that matches their compute, storage, and networking requirements instead of relying on specialized hardware, which can be both expensive and difficult to maintain in most cases. In the context of aerial networks, the types of payloads that UAVs would require to be on-board in order to provide a network service over an aerial network could be reduced by relying on generic hardware instead (which is usually easier to compact in comparison with specialized equipment), which combined with their increased mobility, can lead to the development of novel applications that would be difficult to provide with current solutions. Inside this equipment, the networking services are provided using Virtualized Network Functions (VNFs), software implementations of network functionalities (e.g., a router or a traffic generator). In order to build a complex networking service over a network, it is necessary to interconnect several VNFs in order to create a Network Service (NS), and these VNFs usually expand over several nodes that have the necessary computational, networking, and storage resources for running the service over an infrastructure (in this case, partially or fully composed by the aircraft payloads), denominating it as the NFV Infrastructure (NFVI). Some prominent examples of this combination can be found in works like [26], where authors propose a video-surveillance system for big-poorly Internet covered areas using UAVs, whose mobility allows their distribution along the desired geographical regions, using several VNFs to transmit video through a network deployed inside the devices that provide the service (i.e., the node that is part of the NFVI, which can be either the own aircraft or its payload). Using this combination, authors in [27] propose a softwarization architecture for UAVs and wireless sensors networks, illustrating this architecture with an agricultural example (where several data types regarding temperature, humidity, etc. were obtained through sensors). Other example can be found in works like [28], where the authors present an airborne computing platform design to enhance UAV functionalities. Another example is shown in [29], where we deploy an IP telephony service though a SUAV network using NFV in order to execute the necessary networking elements to generate and distribute data between users, which could be useful in emergency situations and search-and-rescue operations for poorly covered areas.

Although the prospect of this combination is promising, some other works have explored the possible limitations of such technologies over aerial networks, where the connectivity is intermittently available due to both the nature of the communications being performed (i.e., the unreliability of wireless communications) and UAVs battery dependency, whose constant depletion (and subsequent exchange) can disrupt the links established between them. In our previous work [30], we analyzed the different issues that NFV Management and Orchestration (MANO) platforms might face in resource-constrained aerial networks, showcasing that popular solutions like Openstack (Open Source Cloud Computing Infrastructure: https://www.openstack.org/ (accessed on 27 April 2020)) might not be an optimal solution for these networks due to its high technical requirements, transport-layer limitations and its centralized approach for the management communications. Openstack is an Open Source cloud operating system in charge of managing the computational, networking and storage resources from an NFV infrastructure for the deployment of VNFs, i.e., it is a Virtualized Infrastructure Manager (VIM). In order to perform this resource control, it uses a set of services for each one of its functionalities (i.e., one service is in charge of the networking resources, other for allocating computational resources, etc.), controlling them from a central node, the controller. However, all these services are generally resource demanding, which is not suitable for resource-constrained environments or intermittently-available nodes, and due to its a cloud-oriented approach it focuses on supporting Virtual Machines, and such environments with limited computational resources might not be able to efficiently use this type of virtualization for its higher resource cost.

In consequence, there has been an effort on proposing more lightweight alternatives that could both be applied in systems with lower resources or cloud environments to increase their performance without compromising their functionalities: container technology. This approach does not isolate the Operating System (OS) of the host through hypervisor technology, but rather modifies the underlaying host OS in order to separate the instances through kernel namespaces and control groups. In consequence, containers provide better performance when compared with VMs, as they require a lower amount of resources to perform the same functions [31,32]. In this context, we can find the open-source solution Kubernetes [33], also known as K8s, as one of the most popular platforms in both industry and academia. K8s is an open-source solution to deploy and manage applications based on Docker (Docker: https://www.docker.com/ (accessed on 27 April 2020)) containers allowing the managing of workloads and services, as well as their automatic configuration and deployment. Thanks to its ever-growing support due to its large community of users and developers, some researchers have already proposed solutions combining K8s with UAV technologies, as can be seen in works like [34], where authors propose a cloud-based platform for a control system architecture in order to allow controlling several UAVs despite their physical location, Ref. [35] where authors propose a cloud-native NFV-driven Intrusion Detection System using Kubernetes, and Ref. [36], where authors present an architecture to support reconfigurable multimedia services for a practical emergency environment use case of rescue operations using UAVs. Unfortunately, K8s is a resource-demanding solution since it is still cloud-oriented (i.e., its application in large data centers with full connectivity where connectivity drops are infrequent), which limits its application in further resource-constrained environments such as IoT environments. Hence, a lightweight version of Kubernetes called K3s (Lightweight Kubernetes: https://k3s.io/ (accessed on 27 April 2020)) is currently being developed as well, reducing the K8s footprint and size for its suitability in resource-constrained environments, as authors in [37] propose using this platform to incorporate IoT devices into a smart city.

Other approaches have been proposed to deal with MANO communications in resource-constrained environments by improving their performance over “cloud-oriented” solutions, particularly the Eclipse Fog05 Fog Infrastructure Manager (FIM) (Fog05: https://fog05.io (accessed on 27 April 2020)). Fog05 is an open-source project with the objective of deploying a decentralized infrastructure to provide and manage all the compute, storage, communications, and I/O resources from a network. One of the main advantages of this platform is its decentralized approach to resource orchestration, as it eliminates the necessity of having a central controller unit to coordinate the management between nodes, effectively distributing the orchestration across the network itself. Moreover, Fog05 has a very reduced footprint and wire overhead, allowing its use on severely resource-constrained nodes (usually present in Internet-of-things (IoT) environments), as it also relies on the Zenoh protocol (Zenoh: http://zenoh.io (accessed on 27 April 2020)) to unify data in motion, data in-use, data at rest, and computations in a network. This protocol is considered the evolution of the well-known Data-Distribution Service (DDS) protocol [38], which has seen limited implementation in UAV-oriented scenarios [39] due to its reliance on multicast technologies, which present notorious performance problems under those conditions [40]. However, Zenoh improves this behavior by blending the publish/subscribe model with geo-distributed storage, allowing the nodes to retrieve this information at any moment from other nodes spread throughout the network, and not only when the information is being published. Nevertheless, this platform is currently under development, so it has seen limited support at the time of writing this publication, with few examples of its application such as the one seen in [41], where authors implemented a system to provide a 360-video streaming service end-to-end across three computing tiers (cloud, edge, and constrained fog).

## 3. Testbed Components Description

Until now, UAVs that have been deployed forming an aerial network are typically controlled using an RLoS link. Therefore, a GCS (in charge of UAV management) should be geographically adjacent to the UAVs in order to properly establish this link for its management. In consequence, as each GCS manages its own set of UAVs, the coexistence of several UAS in the same airspace is not feasible (i.e., if a UAS is operating in relative proximity to other UASs without considering the operations performed by the other UAS, the risk of having an accident is logically high).

For the correct establishment of next-future UAV applications, UASs must be connected to the UTM regardless of the technology chosen for this connection, which can be separated into three different alternatives: direct connectivity (e.g., packet network), 3GPP cellular connectivity, or satellite communications. This UTM manages the airspace, ensures safe operations, and computes the aircraft flying trajectories to avoid collision and accidents. The interconnection of all the elements encompassing a UAS with the UTM opens the BRLoS UAS management paradigm, producing a more automatic and agile deployment in comparison with legacy UAS scenarios and possibly allowing for an integrated airspace.

**Figure 2:**
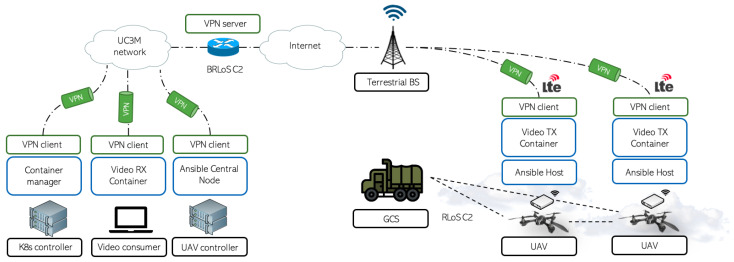
High overview of the proposed testbed.

As can be appreciated in Figure 2, this article presents a testbed to experiment with new UAV services and applications managed by both BRLoS and RLoS models. However, all the experiments and efforts have been designed to properly establish the BRLoS management (RLoS models have been studied in our previous work [10,42]). To this purpose, both the GCS and UAVs are connected to a UAV-controller (in this case, also acting as UTM) using the regular 4G/LTE cellular network. The following subsections describe the communication architecture, the automated UAV control system, and the virtualization platform, including the devices and technologies chosen for its design.

### 3.1. Cellular-Assisted UAV Communications

**Figure 3:**
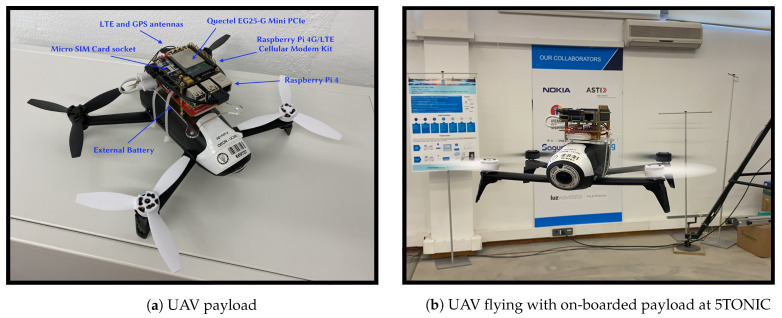
Details of UAV payload, and UAVs in flight.

To support research activities, this publication proposes a novel communication model for this testbed that can be on-boarded as the payload of almost any UAV (we have used small UAVs to verify this fact). This prototype incorporates a Raspberry Pi 4 (RPi) (Raspberry Pi 4 Model B: https://www.raspberrypi.org/products/raspberry-pi-4-model-b/ (accessed on 27 April 2020)) Single Board Computer (SBC). The RPi can be equipped with one of these two alternatives to connect with the cellular network: (i) the Sixfab 3G/4G& LTE Base HAT (Raspberry Pi 4G/LTE Cellular Modem Kit: https://sixfab.com/product/raspberry-pi-4g-lte-modem-kit/ (accessed on 27 April 2020)), or (ii) a 5G/4G/3G HAT (SIM8200EA-M2 5G HAT: https://www.waveshare.com/sim8200ea-m2-5g-hat.htm (accessed on 27 April 2020)). The RPi acts as the brain of the UAV, having the ability to host different network designs and configurations to enable its communications, i.e., this device allows the use of any protocol or communication solution to establish communications with other devices or/and the Internet, for example implementing a Virtual Private Network (VPN) tunnel. Moreover, the HATs provide the RPi with a simple interface bridge between mini PCIe cellular modems. Three different modems have been selected for this system: the Telit LE910C1 Mini PCIe LTE CAT1 Module (Telit LE910C1 Mini PCIe LTE CAT1 Module: https://sixfab.com/product/telit-le910c1-mini-pcie-cat1-lte-module/ (accessed on 27 April 2020)), the Quectel EC25 Mini PCIe 4G/LTE Module (Quectel EC25 Mini PCIe 4G/LTE Module: https://sixfab.com/product/quectel-ec25-mini-pcie-4g-lte-module/ (accessed on 27 April 2020)) (both compatible with the Sixfab HAT), and the SIMCom SIM8200-M2 (compatible with the Waveshare HAT), allowing its straightforward configuration to start using cellular internet with the RPi. All equipment is powered with a portable battery of 3.7V and 3800 mAh. This equipment can be appreciated in Figure 3a.

Despite establishing connectivity with the commercial cellular network, communications cannot still be enabled between the UAV-controller and the device since the HATs assign a private IP address to the RPi, independently of the IP address that the PCIe modem uses to connect to the network (provided by the commercial operator). In consequence, all the communications between the RPi and the exterior (i.e., Internet) are performed using a Network Address Translator (NAT) (included in the HAT). By extension, the RPi can communicate with the UAV-controller (as the UC3M network provides public IP address), but not in the reverse direction, as private IP addresses are not directly reachable from the Internet (i.e., the NAT is preventing the UAV-controller from reaching the RPi). Unfortunately, the HATs do not allow port opening or port forwarding configuration.

In order to solve this issue, since the UAV-controller must be able to communicate with the RPi, the testbed will utilize a VPN service where all the UAS equipment is connected in order to enable a relay service. This VPN allows the communication between both elements thanks to the encapsulation, and subsequent routing, of the information that has to be sent. Furthermore, the VPN service provides additional advantages that are beneficial for the testbed infrastructure. For instance, since any UAV is able to change the BS (as a standard ground user equipment) due to the inherent UAV mobility, its IP address might change when this movement is performed. However, by using the address space provided by the VPN service, these handovers should be transparent for the UEs as long as the UAV has connectivity to the Internet, because the VPN will deal with the routing since the device will not modify its VPN IP address, even though its IP address (the one providing Internet to the RPi) might change. Moreover, using a VPN provides an additional layer of security, as all the communications between its elements will be encrypted to allow its transmission through the VPN tunnel, preventing man-in-the-middle attacks or the unauthorized attachment of malicious nodes to the network. Details on including data encryption, such as VPN service, can be found in our previous work [43].

### 3.2. Automated UAV Control System

In order to support UAV experimentation activities in flight, this paper introduces and implements a system to control UAV trajectories automatically. It is essential to have a centralized system capable of simultaneously controlling several UAVs to facilitate flight operations and reducing system complexity. In the proposed solution, all UAVs can be operated from a GCS (RLoS) or from the UAV-controller (BRLoS). This procedure has been completely automated by transferring the trajectories from the UAV-controller (which is the entity in charge of the computation of such trajectories) into the RPi (in this case, located as the UAV payload) to be executed later in the UAVs. The UAV-controller is in charge of transferring the optimal trajectories to all UAVs in the scenario to avoid collisions with possible obstacles and other UAVs coexisting in the same airspace. A UTM development (meeting all the requirements mentioned above) will replace the UAV-controller functionality. Further research in this direction can be found in the literature [44,45,46].

The automation of this service is based on the Ansible platform (Ansible platform: https://www.ansible.com/ (accessed on 27 April 2020)). Ansible is a software tool that automates software provisioning, configuration management, and application deployment. Moreover, it does not require third-party applications to establish communication channels between the central node (UAV-controller) and the hosts (UAVs), as it uses the well-known, and extensively used, SSH protocol.

On the other hand, UAVs commonly have an Application Programming Interface to control them. This procedure is performed from the RPi (UAV payload) which has previously received the trajectories. One of the most popular examples is the MAVlink library [47]. MAVLink is a lightweight protocol to communicate not only with UAVs, but also with on-board UAV components. Another representative example is the Robot Operating System (ROS) [48], which is a set of building robot applications that are also employed to control UAVs. Using the specified protocols instead of proprietary protocols proffers the system huge flexibility and versatility since they have a big developer community, and in consequence, several commercial compatible UAVs become available. In our case, an open-source Python library called pyParrot (pyParrot’s documentation: https://pyparrot.readthedocs.io/en/latest/ (accessed on 27 April 2020)) (compatible with a great variety of Parrot UAVs) is used to send the instructions from the RPi to the UAV. The instructions are simple commands already implemented in the library (e.g., take-off, landing, turn right, turn left, etc.) for these experiments. However, these instructions may become more complex if the received trajectories from the UAV-controller requires to. The instructions are sent to the UAV through its WiFi interface. These instructions can be transmitted using various alternatives, such as the serial port, the USB port, or another communication channel. However, the available UAV in the testbed (Parrot Bebop 2) can only be commanded using WiFi.

### 3.3. Virtualization in UAVs: The Power of Containers in Aerial Networks

As explained in Section 3.1, UAVs will carry as their payload RPi devices in order to execute their movement instructions (sent from a remote GCS/controller), provide services to the users of the aerial network, such as those mentioned in Section 2 like rural monitoring, or execute specific applications depending on the mission type that has to be carried out. However, RPIs are generally considered resource-constrained devices in the context of NFV environments, since the amount of computational resources that are available to the device are considerably lower as compared to other equipment types such as Mini-ITX or servers. In consequence, it is desirable to save as many resources as possible when providing services and/or running applications in order to increase the available space to run new functionalities and/or scaling up operations without having to increase the number of devices in the network. Moreover, battery constrains are also a source of issues in aerial networks since RPis are forced to use a finite (and small) battery that will drain over time until its depletion, so consuming less resources can increase their operative life-time before requiring a substitution [49]. However, hypervisor-based solutions like Virtual Machines (VM) (frequently used in NFV) are computationally intensive, as they need to imitate a full physical infrastructure to provide a single OS with its own kernel. Therefore, containerization can be regarded as a more adequate solution in these situations, since they run over the host Operating System to build its necessary components through kernel instructions, saving computational resources, as no hypervisor is required for the operation. Hence, aerial networks can benefit from container technology to save resources (and potentially battery life-time), making them good candidates for their implementation for service provisioning. Nevertheless, in order to build an NFV-driven (or close) solution, it is mandatory to have a control plane able to manage and orchestrate all of the containers needed for the softwarization of an application or network service, including their instantiation, life-cycle management, and logical connectivity. In this context, the Kubernetes tool (i.e., K8s) can be considered as an adequate solution for these tasks.

As it has been previously described in Section 2.2, Kubernetes is an open-source system for the automatic deployment, management, and scaling of containerized applications. This platform allows its users to easily deploy container images inside a cluster, which can be composed of several hosts that provide the infrastructure and resources to run the containerized applications. This container orchestrator provides several functionalities that assist the deployment of applications inside an infrastructure, including the automatic rollouts and rollbacks, ensuring that newer versions do not leave the application unavailable; self healing abilities, achieved by restoring or re-deploying containers that failed during the execution of an application; IPv4/IPv6 dual stack, horizontal scaling, which can even be automated based on CPU usage, among others. All these functionalities can be achieved thanks to its reliance on container technologies: K8s uses different container runtimes (particularly containerd (Containerd: https://containerd.io (accessed on 27 April 2020)), CRI-O (CRI-O: https://cri-o.io (accessed on 27 April 2020)), and Docker) to instantiate container-based applications, which are usually lighter than VMs since they are built on top of the kernel of an OS (contrary to hypervisor-based solutions where the software fully imitates the physical infrastructure where the VM is running).

In order to perform any kind of deployment inside a K8s infrastructure, it is mandatory that two components are present, and properly linked, between the hosts and/or the machines building the infrastructure, establishing a cluster. It is not mandatory for these machines to be in the same physical location and/or to have direct connectivity between them through a single LAN or subnet. However, it is necessary to have full networking (i.e., layer 3) connectivity between all the components of the cluster, as it is specified by K8s documentation. The essential components of every K8s cluster are the following ones:

- Controller-plane: this component is in charge of the management and orchestration of the K8s cluster and the applications running inside. Its main tasks include the instantiation of pods (minimal unit where containers have to be deployed) & services, and react to cluster events such as scaling up/down a deployment, managing cluster errors, pod re-deployments, etc. This control-plane unit, commonly referred to as the “master” node, is usually deployed in one single host, although it can be deployed across multiple machines if necessary.
- Worker node: these nodes are in charge of providing the resources to the cluster. Inside these nodes, pods will be deployed to run the applications in the Kubernetes cluster, maintaining their functionality and reporting their status to the master node. In every cluster, there must be at least a single worker node, and they are not limited to a single host, i.e., inside a host there could be multiple workers (for example, multiple VMs).

Kubernetes is available for a wide array of Operating Systems such as Ubuntu 16.04+, CentOS 7+, or Fedora 25+, as well as different architectures like amd64 and arm64, this last one being typically used in mobile terminals and small devices such as RPis. Therefore, K8s is regarded as a flexible platform suitable for its use in multiple use cases that require an heterogeneous environment with multiple types of devices, including small equipment that can be on-boarded in most UAVs due to its small size and lightweight nature. This fact combined with the lightweight nature of containers compared to traditional virtualization solutions (i.e., VMs) assists in the development and deployment of network services on top of UAVs, including one of their most popular applications in their current form: video transmission.

All the aforementioned functionalities and advantages have been decisive for choosing this tool as the container manager for the implementation of the current testbed depicted in Figure 2. As a consequence, the RPis carried by the UAVs and the video-consumer will become the computing nodes of the infrastructure (using K8s’s terminology, worker nodes). A K8s controller (i.e., a master node) manages the instantiation, management, and termination of all the containers of an application deployed in the infrastructure. Communications between these components are entirely performed through the VPN service, ensuring both secure transmission (e.g., a man-on-the-middle attack) and preventing unauthorized users from joining the infrastructure (e.g., a malicious UAV posing as a legitimate computing node of the platform). In the particular case of the testbed, the UAVs will host a video transmission application (Video TX Container in Figure 2).

Video transmission has been one of the main use cases of UAVs since their inception. Thanks to their advanced mobility, these aircraft are able to on-board different types of cameras to take pictures and/or video footage. Moreover, some models are shipped with one, or several, integrated cameras by default as a consequence of its high demand in both industrial and commercial environments for a wide variety of applications such as video filming, wild life monitoring, surveillance, or search and rescue operations. In consequence, this testbed will introduce UAVs able to generate and send video traffic to a particular machine or client, either localized inside the testbed (video-consumer in Figure 2) or outside. This application will be deployed as a single container running in the worker nodes located at the RPi, where the video traffic will be generated. These nodes are represented as the UAVs in Figure 2. Afterwards, this video will be sent to a specific location, either to nodes located inside the cluster (e.g., the video-consumer) or located outside the cluster (e.g., a PC at a remote location). If the UAV goes offline at any moment in time, either due to a battery replacement cycle or connectivity issue, K8s will try to instantiate this application in a new UAV or, if no others are available, wait until one of them is available to re-deploy the container again, showing the potential of K8s to reduce service cut-offs in aerial networks.

## 4. Testbed Experimentation

### 4.1. Communication Performance Evaluation

In this section, the UAVs communication performance has been evaluated to guarantee that it meets the requirements defined by the 3GPP in Release 15 (TR 36.777) [50]. These requirements are based on three network parameters: (i) data rate, which should be set between60 and 100 Kbps for command and control in both downlink and uplink; (ii) reliability, which should be below 10^−3^ of packet error rate in both downlink and uplink; and (iii) latency, which should be below 50 ms (100 ms of RTT) in the downlink. It should be noted that these KPIs are related to management assignments (C2). Each particular service will have its individual KPIs (for example, real-time video streaming KPIs are quite different).

We have first tested the quality of the Internet connection with the well-known Ookla speed test (Ookla speed test: https://www.speedtest.net/ (accessed on 27 April 2020)). The speed test uses public servers to perform a quick and realistic diagnosis of Internet connectivity. In this context, the network performance was also tested from the UAV upon the UAV-controller since, in practical experimentation, the minimum values have to be met against this entity. Other limiting factors may arise in the second experiment, such as using a VPN service (since all the traffic should traverse the VPN server).

Experiments have been replicated with the three available mini PCIe and a commodity smartphone (Xiaomi Redmi Note 9S). The purpose of repeating the same experiments employing different devices is to determine if the potential limiting factors are procured from either the cellular network characteristics, e.g., the status of the network, the antennas used, the strength of the signal at certain points of time, or the devices themselves (i.e., checking if the devices could be limiting the quality of the connection due to a hardware/software limitation). Likewise, experiments have been conducted using two different Internet Service Providers (ISP) to check if connectivity performance varies depending on the network operator. The selected ISPs are Movistar and Yoigo (the first and the fourth operator in Spain ranked by the number of users). Twenty repetitions of the experiment have been conducted at different time intervals randomly distributed throughout the day to ensure that the external effects are not reflected (or at least mitigating their effect as much as possible) in the results. The main purpose of these measurements is not to characterize the network, as this would require an extensive set of measurements with varied coverage areas and having an insight of the network infrastructures involved to rule out any other factors having an effect on the results achieved. The main purpose instead is to verify whether the results fulfill the 3GPP KPIs.

**Figure 4:**
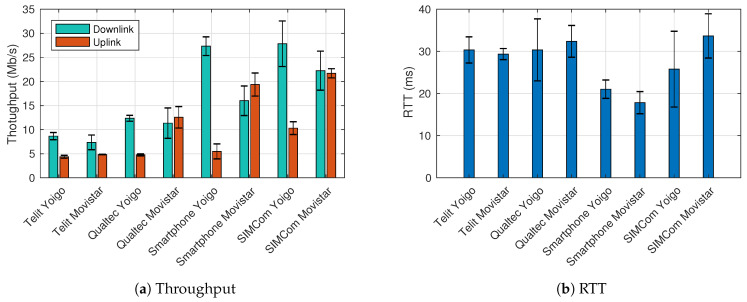
Performance analysis using Ookla speed test.

As it can be appreciated in Figure 4a both downlink and uplink are considerably limited for the Telit mini PCIe due to its technical characteristics, which define a nominal rate of 10 Mb/s in downlink and 5 Mb/s in the uplink, regardless of the selected ISP. On the other side, experiments with Qualtec mini PCIe should not be so limited since its nominal rate is 150 Mb/s and 50 Mb/s in downlink and uplink, respectively. However, after comparing with the smartphone and SIMCom PCIe results, both the downlink and uplink appear to be limited despite its technical characteristics (i.e., it has been demonstrated with the SIMCom results that the performance of the network is superior to that obtained by Qualtec PCIe). Finally, the SIMCom PCIe results improve the results obtained with the smartphone, which can be considered the benchmark. It should be noted that the Waveshark HAT, in combination with the SIMCom modem, has a notably higher price than the rest of the selected equipment, so we can infer that the performance is better because it belongs to an upmarket product. On the other hand, RTT experiments (Figure 4b) reveal that all devices and ISPs fulfill 3GPP KPI requirements. Please, take note that the error bars correspond to the standard deviation.

This experiment also reveals that the Movistar network has balanced downlink and uplink data rates; meanwhile, Yoigo prioritizes downlink data rates. For selecting a final prototype, it is important to consider that UAVs potential applications will overload mostly the uplink (i.e., the highest data rate will be generated on the UAV and sent in the uplink). However, all the combinations (mini PCIe and ISPs) comply with the 3GPP KPIs.

**Figure 5:**
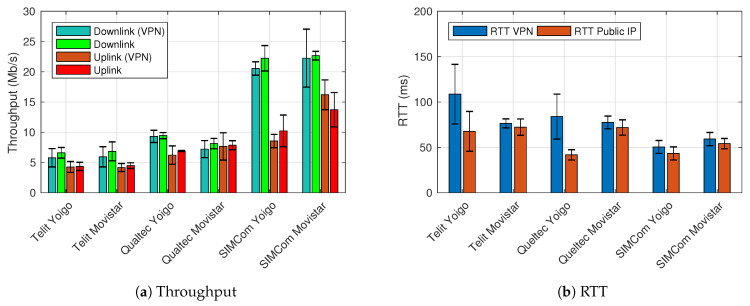
Performance analysis upon the UAV-controller.

In the second set of experiments, we have measured the performance from the RPi upon the UAV-controller, enabling and disabling the VPN service to quantify the performance penalty that the VPN may introduce. Throughput results are similar in the second experiment to those obtained with the Ookla speed test as represented in Figure 5a. As expected, there is a slight decrease in the performance since data packets are not only traversing the VPN server but also have to include the VPN headers (decreasing the data payload size). However, the values are still much higher than those required for C2. On the other hand, the RTT results (Figure 5b) are considerably worse, although they are within the requirements set by the 3GPP KPIs. This phenomenon occurs because of the VPN encryption and decryption process. The VPN relay is not greatly affecting the performance since the VPN server is located on the same network as the UAV-controller. More details about the performance decrease of introducing a VPN service can be found in [43]. Reliability tests were also performed by sending a 200 Kb/s stream as required in the KPIs (both downlink and uplink), and no packets were lost during this process.

It can be concluded that all the devices fulfill the management requirements. Other services can be provided using this communication solution, such as transmitting periodic High-Definition (HD) images or real-time video streaming (HD 720p). Still, some challenges may arise when using this technology for complex multimedia applications or multiple services simultaneously.

### 4.2. 5G Standalone Benchmark

By December 2020, Ericsson has activated 5G SA technology at 5G Telefonica Open Network Innovation Centre (5TONIC) [51]. 5TONIC is a leading research laboratory focusing on 5G technologies based in Madrid, where members from both industry and academia work together in specific research and innovation 5G experimentation projects. The deployment of 5G SA in this laboratory is a crucial technological milestone for validating the solutions developed by 5TONIC verticals partners allowing promising capabilities enabled by 5G technology demonstrations. This deployment includes the integration of the Ericsson Radio Access Network (RAN) [52] and the Core solutions for 5G NR standalone [53] in the laboratory facilities. With this achievement, 5TONIC becomes one of the first open research and innovation laboratories in the world to offer 5G SA capabilities to validate vertical applications.

Due to the lack of commercial deployment of this technology, the 5TONIC 5G SA network has been used to benchmark the network performance offered by a real 5G SA deployment. Although these measurements have been conducted in a laboratory environment with ideal conditions (compared to Section 4.1 measurements), they demonstrate the upcoming 5G SA technology potential.

The NETGEAR CPE (Nighthawk M5 5G WiFi 6 Mobile Router: https://www.netgear.com/home/mobile-wifi/hotspots/mr5200/ (accessed on 27 April 2020)) was used to perform these experiments. This device connects through the Ethernet interface with the RPi to provide connectivity. Thanks to its compact size (10.5 cm × 10.5 cm × 2.15 cm) and low weight (240 g), this device can be on-boarded on almost any UAV, as shown in Figure 3b. Moreover, it includes a rechargeable battery promising battery life-times for one day at total operation. As in the previous section, the Ookla speed test has been used to diagnose Internet quality. We obtained an average value of 387.98 (±90) Mb/s in the downlink and an average value of 52.3925 (±5) Mb/s in the uplink. RTT results are 11.94 (±3.5) ms. These results vastly improve the ones obtained by a commercial 4G network. Accordingly, this technology has to be contemplated for the near future and can be considered as the enabling technology to deploy the full potential of UAV networks. However, an exhaustive performance evaluation will have to be carried out with commercial deployment.

### 4.3. Indoor Flight Validation

Indoor flight tests have been carried out to validate the payload as an operation onboarded system (no outdoor flight has been done due to regulatory restrictions). These results serve as a proof of concept demonstrating the functionality of the proposed testbed. For this purpose, indoor tests have been carried in the 5TONIC laboratory. On the other hand, the K8s controller, Video-consumer, and UAV-controller elements (see Figure 2) are located at UC3M. In this way, an inter-site experiment is performed to demonstrate that the BRLoS management operations run correctly. As it can be appreciated in Figure 3a,b, the communication prototype (RPi + Qualtec PCIe) has been on-boarded as an UAV payload, using a compact battery to power up the prototype. The overall dimensions of this prototype are 6 × 11 × 4 centimeters, with a total weight of 200 g including the battery. It is essential to consider lightweight and compact equipment since the power required by the UAVs to carry all its load can significantly impact the life-time of the UAV main engines battery.

It is essential to consider that Multi-UAV operations entail certain risks, not only in colliding with aerial traffic (i.e., other UAVs, human-crewed aircraft in the proximity of airports), and accidents including people and vehicles on the ground in populated areas. Authors in [54] proposed a complete risk assessment model for UAV operation in urban environments with three main risk categories (people, vehicles, and human-crewed aircraft). The mission planner generally contemplates the operations and factors involving the most significant risk. However, unbounded exploration can lead to undesirable scenarios. To solve that issue, authors in [55] propose a framework that evaluates the risk of the potential actions. In case of actions deemed too risky, they are replaced with another action of lower risk.

**Figure 6:**
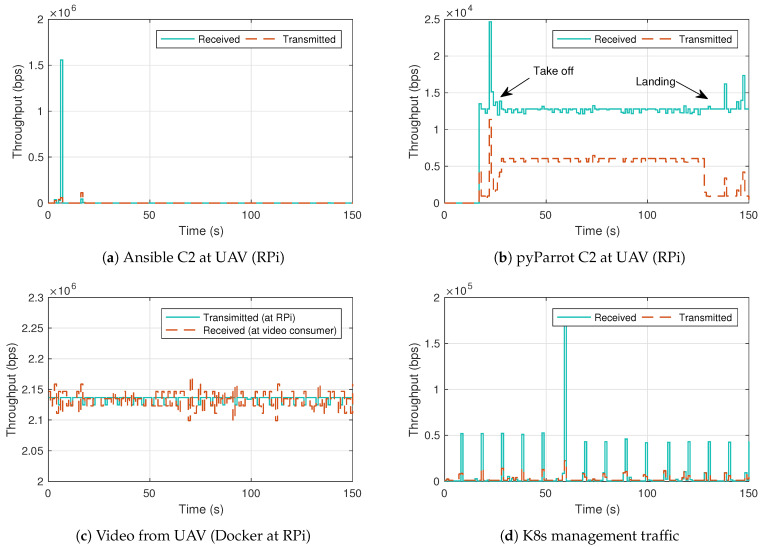
Measurements in indoor flight.

C2 information has been transmitted from the UAV-controller to the RPi (UAV payload) using the Ansible automation platform explained in Section 3.2. Once the payload has received the trajectory, it replicates the instructions to the UAV using its WiFi interface (also explained in Section 3.2). This traffic is represented in Figure 6a,b. Figure 6a shows a traffic peak corresponding to the trajectory transfer. On the other hand, Figure 6b displays the transmitted and received traffic between the RPi and the UAV. The two traffic peaks that can be appreciated correspond to the take-off and landing procedures.

On the other hand, the testbed virtualization platform has been used to test multimedia services viability. A docker container deployed on the RPi (Video TX container in Figure 2) transmits a 2Mb/s UDP traffic flow to a docker container deployed on the video-consumer. This stream emulates an HD video transmission (HD 720p [56]), a target rate that most HD cameras are able to generate. All this traffic has been sent using the VPN tunnel described in previous sections, since it provides an extra security layer to the information being transmitted (i.e., preventing third-party attacks that could compromise the video feed in real scenarios). Figure 6c reveals that the video-consumer has correctly received the flow.

Finally, the traffic transmitted and received for the correct management of the virtualization platform is shown in Figure 6d. This traffic should not be neglected as it shares the same channel as other critical communications such as C2, and its presence can disrupt other traffic if it requires a high amount of bandwidth for its proper functionality. However, it is relatively small in comparison to the available bandwidth of the channel, with an average of 2.71 Kb/s for transmitted management traffic and 6.52 Kb/s for received management traffic (representing less than 1% of the available throughput), so it is unlikely that this traffic will interfere with C2 communications and other applications embedded in the UAV payload.

### 4.4. Indoor Flight with Connectivity Loss

**Figure 7:**
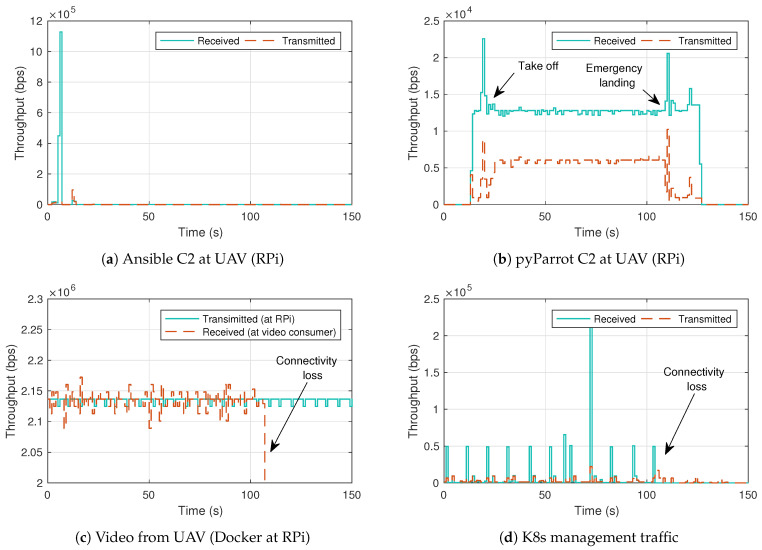
Measurements in indoor flight with connectivity loss.

Once the feasibility of the testbed was demonstrated in the previous Section 4.3, the experiment is repeated with the introduction of a connectivity loss between the UAV and the UAV-controller. UAVs must have some degree of autonomy when communications are lost with the UTM in order to avoid accidents or collisions with other aircrafts, since losing connectivity with this element could be disastrous for the UAV if no alternative method to calculate its flying trajectory is available, and it is important to have in mind that communications depend on an external network that can introduce errors and temporary disconnections to the UTM. In order to reproduce this situation, the interface that provides connectivity to the RPi is switched off. Figure 7c,d shows that the connectivity is lost around second 110.

In the meantime, the RPi is continuously monitoring the connectivity with the UAV controller. After detecting a connectivity loss, it waits for a predetermined time (set through a programmable parameter) to check if the communications have been recovered. As it is not the case, the UAV performs an emergency landing routine, as shown in Figure 7b. This instruction can be modified by any other procedure, such as a return to home (RTH) instruction, or remain static (hovering) in a certain position waiting for new communications from the UTM. However, it is essential to point out that the UAV must have some autonomy in this decision-making process, ensuring that all these instructions can be safely performed and, in addition, using detect and avoid mechanisms to evade obstacles while executing these instructions.

## 5. Conclusions and Future Work

This paper presents as the main contribution a practical cellular-assisted UAV to enable BRLoS traffic management in the context of the Labyrinth project. This testbed will be employed to conduct validation analyses within the project.

Different experimentation using this testbed has also been presented: on the one hand, it has been demonstrated that the selected communication equipment and the testbed design fulfill their purpose and meet the KPIs set by 3GPP for a proper BRLoS operation. However, the results reveal that the 4G commercial network may have some limitations for applications beyond C2, while the conclusions obtained from the 5G SA benchmark reveal promising results in terms of bandwidth and latency. On the other hand, indoor flight experiments have been carried out to guarantee the platform’s viability and functionality.

Besides, the testbed incorporates a virtualization platform to deploy a wide variety of experiments over the network. This platform improves on traditional virtualization platforms due to its use of containerization technologies, a more lightweight solution for virtualized environments, making its implementation suitable in an environment with resource-constrained devices and/or intermittent connectivity.

This work serves as a starting point for several lines of work and research. First, it would be essential to conduct performance measurements using 5G cellular networks once the commercial deployments are extended. Although 5G characteristics are promising, it will be necessary to exhaustively analyze its output performance to illustrate its potential strengths and weaknesses. On the other hand, it would be interesting to include energy consumption measurements about the communications prototypes. In resource-constrained environments like UAVs, where a battery supplies power to the on-board equipment, it is crucial to properly quantify power consumption in order to calculate its impact on battery life-time (which is very low due to the high energy consumption demanded by flight engines).

## Data Availability

Not applicable.

## Abbreviations

The following abbreviations are used in this manuscript:

UAS	Unmanned Aircraft System
UAV	Unmanned Aerial Vehicle
FAA	Multidisciplinary Digital Publishing Institute
UTM	Directory of open access journals
ATM	ATM
RLoS	Radio Line-of-Sight
BRLoS	Beyond Radio Line-of-sight
C2	Command and control
5G	Fifth-generation mobile technologies
SA	Stand Alone
3GPP	The 3rd Generation Partnership Project
KPI	Key Performance Indicator
SESAR	Single European Sky ATM Research
FANET	Flying Ad-hoc Network
D2D	Device to Device
MANET	Mobile Ad-hoc Network
NFV	Network Function Virtualization
SUAV	Small Unmanned Aerial Vehicles
FIM	Fog Infrastructure Manager
DDS	Data Distribution Service
OS	Operating System
ISP	Internet Service Provider
VPN	Virtual Private Network
RTV	Real Time Video
